# A Case of Acute Myocardial Infarction due to Left Main Trunk Occlusion Complicated With Aortic Dissection as Diagnosed by Intravascular Ultrasound

**DOI:** 10.4021/cr212w

**Published:** 2012-09-20

**Authors:** Masashi Okamoto, Tomonori Amano, Shunzo Matsuoka, Hideki Hirai, Kazunori Masuda, Kanta Nakajima, Atsushi Sueyoshi

**Affiliations:** a Department of Cardiovascular Medicine, Uji Tokushukai Hospital, 86 Kasugamori, Ogura-cho, Uji-shi, Kyoto 611-0042, Japan

**Keywords:** Acute aortic dissection, Acute myocardial infarction, Intravascular ultrasound

## Abstract

A 52-year-old man was transferred to our hospital with a sudden onset of severe chest pains. His electrocardiogram revealed ST-segment elevation suggestive of acute myocardial infarction. Emergency coronary angiography showed subtotal occlusion of left main trunk (LMT) with delayed coronary flow. Because intravascular ultrasound revealed a large intimal flap, we diagnosed aortic dissection involving the LMT. After stenting of the LMT, the patient underwent surgical repair of the aortic dissection. Although it is difficult to obtain a correct diagnosis of aortic dissection complicated with myocardial ischemia, we succeeded in diagnosing this rare condition by use of a intravascular ultrasound.

## Introduction

When an acute myocardial infarction is complicated with dissection of ascending aorta, though rare, it can sometimes result in patient mortality [1]. These cases may be difficult to correctly diagnose because the clinical symptoms of acute aortic dissection are similar to those of myocardial infarction. In particular, when a patient’s electrocardiogram shows ST-segment change, the dissection of ascending aorta causing coronary malperfusion can be overlooked. Here, we report our attempts to make the appropriate diagnosis of these conditions using a intravascular ultrasound.

## Case Report

**Figure 1:**
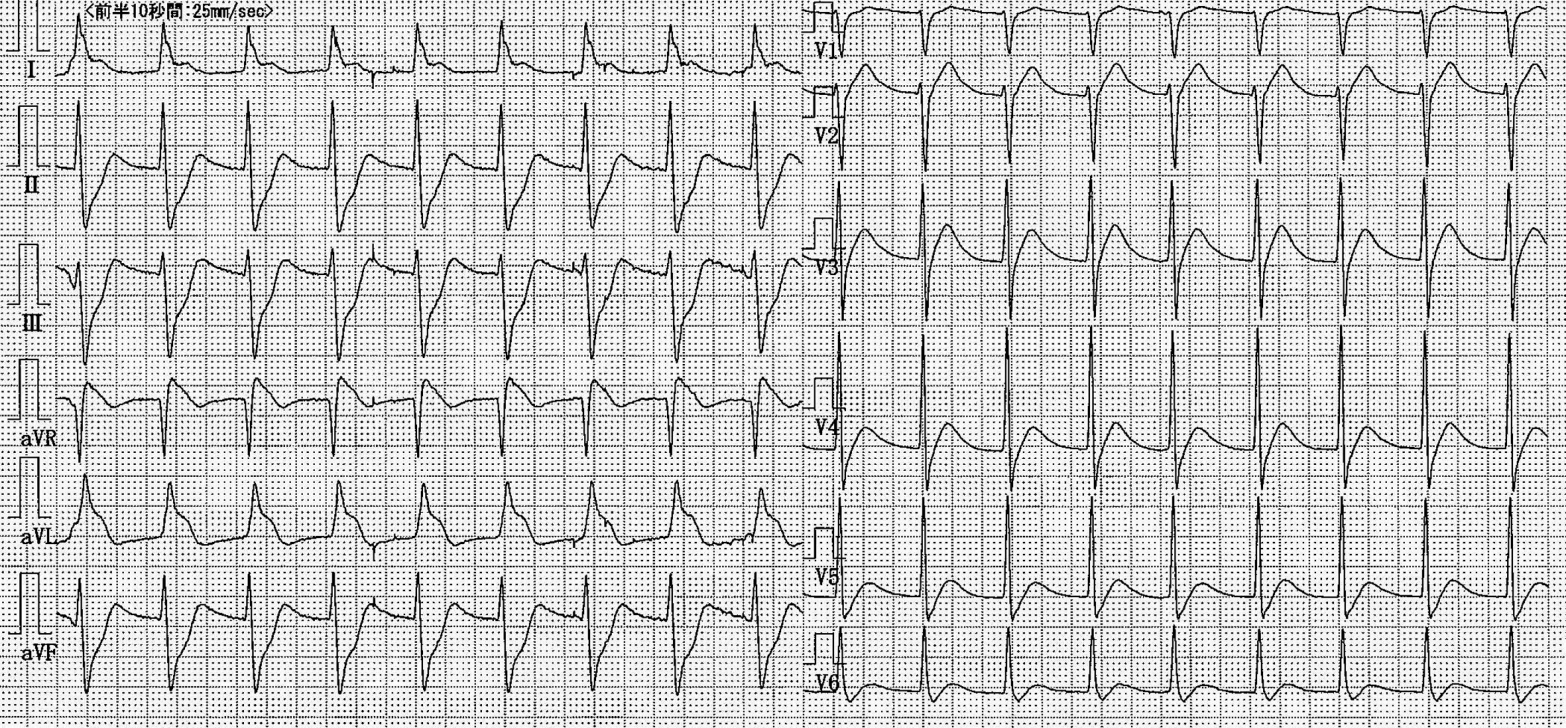
Twelve-lead ECG at admission showed a marked ST-segment elevation in leads I, aVR and aVL, and reciprocal ST-segment depression in leads II, III and aVF.

**Figure 2:**
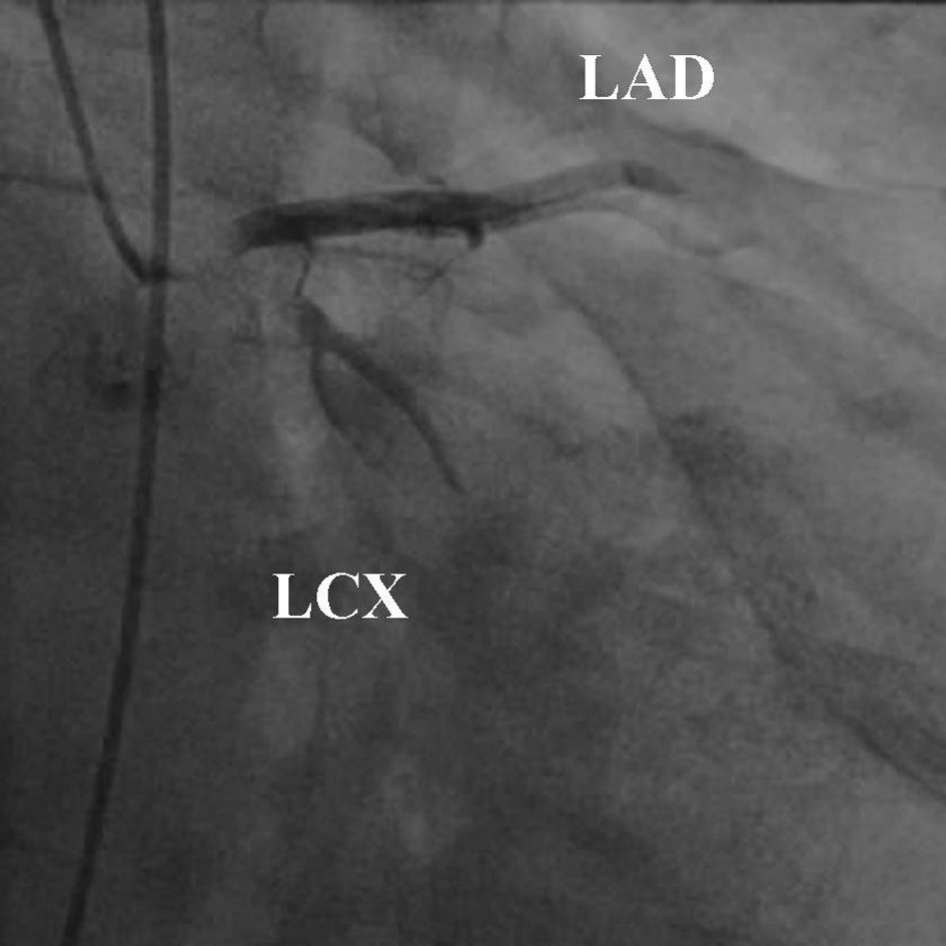
Left coronary angiogram in the right anterior oblique caudal view shows subtotal occulusion of the LMT with delayed coronary flow (TIMI grade 1 flow).

**Figure 3:**
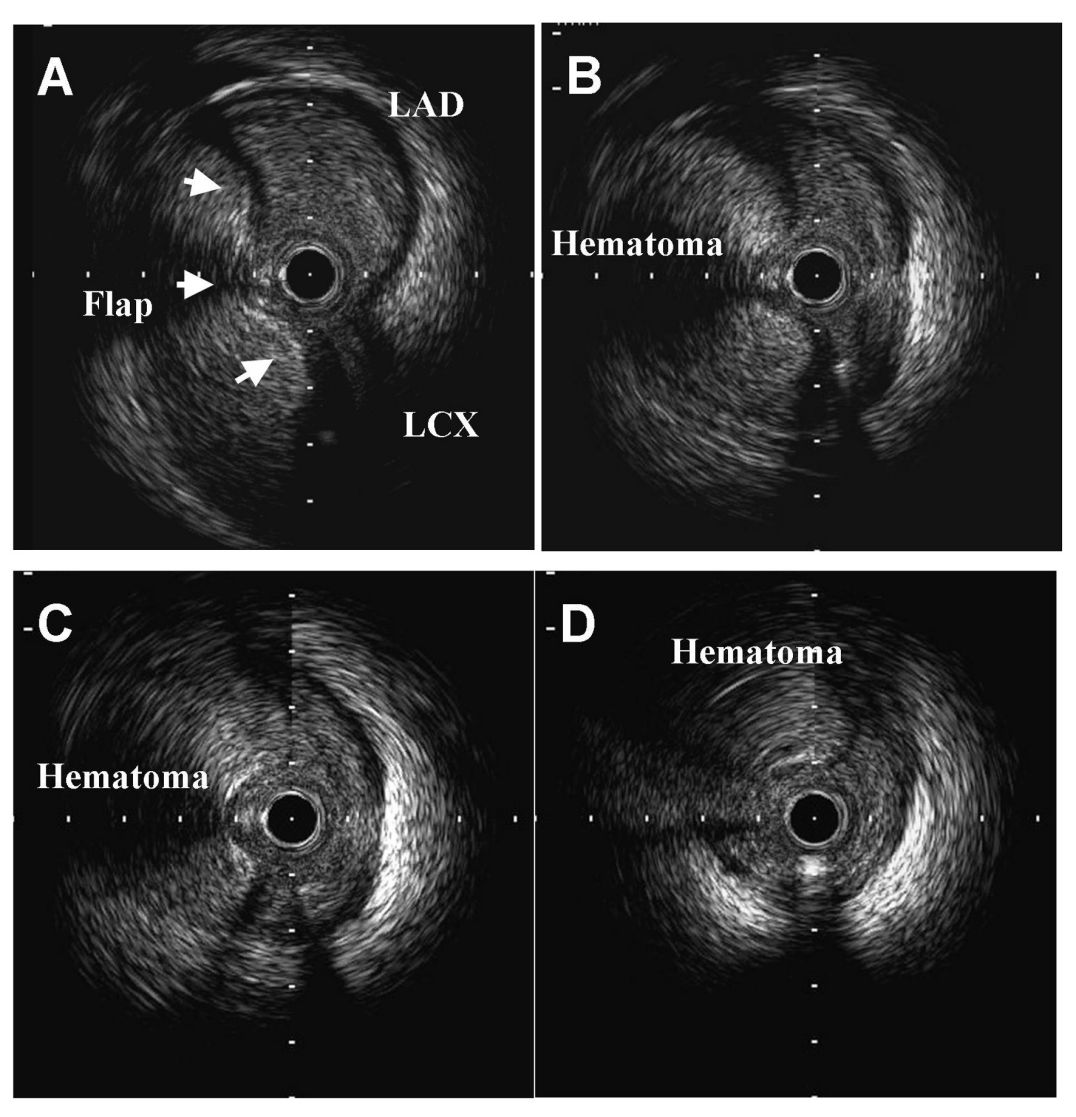
IVUS images at the bifercation of the LAD and the LCX (A), the mid portion of the LMT (B), the proximal portion of the LMT (C), and the ostium of the LMT (D). A large dissecting flap is showen from the ostium to the bifurcation of the LMT (arrowes). The coronary lumen is compressed by a large intramural hematoma.

**Figure 4:**
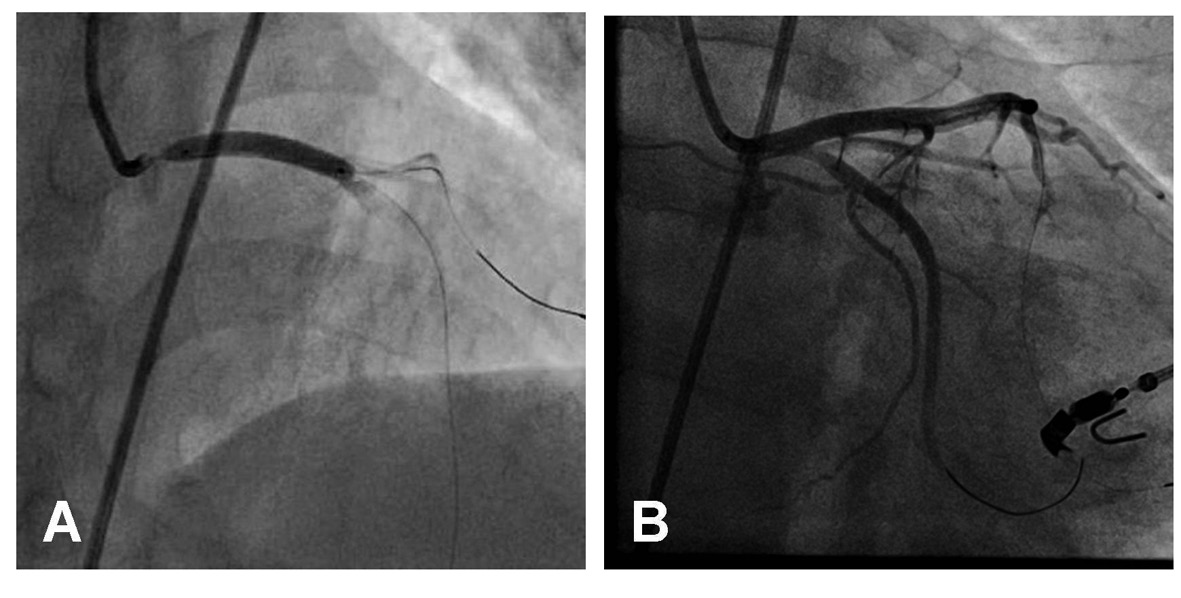
A bare metal stent is implanted in the proximal portion of LAD to the ostium of the LMT (A). After stenting, The coronary flow is improved to TIMI grade 3 (B).

**Figure 5:**
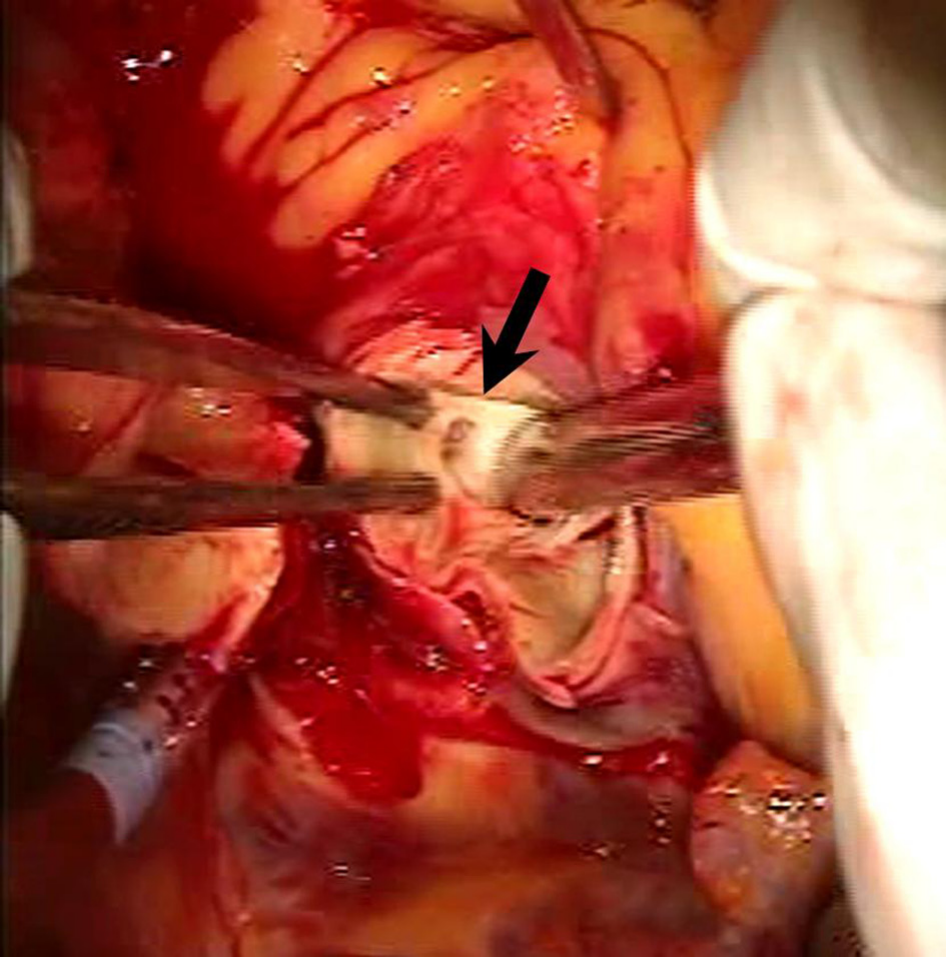
Intraoperative photograph reveals the implanted stent in the LMT.

A 52-year-old man was transferred to our emergency room due to continuous severe chest pains for one hour. On arrival, his blood pressure was 105/66 mmHg, his pulse rate was 106 beats/min, respiratory rate was 32 breaths/min, and oxygen saturation was 91% while breathing 10 liters of oxygen with a non-rebreather face mask. There were coarse inspiratory wheezes bilaterally. Chest radiography revealed bilateral pulmonary edema. The electrocardiogram showed ST-segment elevation in leads I, aVR and aVL, and ST-segment depression in leads II, III and aVF (Fig. 1). Transthoracic echocardiography (TTE) showed diffuse severe hypokinesis of the left ventricle, with no pericardial effusion and no significant aortic regurgitation. Because acute myocardial infarction due to occlusion of the left main trunk coronary artery (LMT) was suspected, the patient was transferred to catheralization laboratory after 17 minutes from his arrival. The results of the emergency coronary angiography (CAG) revealed subtotal occlusion at the LMT with delayed filling of the left coronary artery (TIMI grade 1 flow) (Fig. 2). After two coronary guide wires were inserted into the left anterior descending coronary artery (LAD) and left circumflex artery (LCX), intravascular ultrasound (IVUS) was performed. The IVUS imaging revealed a large intimal flap from the ostium of the LMT to the bifurcation of the LAD and the LCX, suggesting that the ascending aortic dissection had occurred and extended to the LMT (Fig. 3A-D). Repeated TTE testing showed an undulating intimal flap in the proximal aorta, confirming ascending aortic dissection. After the IVUS, acute hemodynamic deterioration occurred. The patient was intubated and a percutaneous cardiopulmonary support system was inserted from his right femoral artery and vein. As a lifesaving procedure, direct stenting of the LMT was performed. We implanted a stent in the proximal portion of LAD to the ostium of LMT, and as a result the coronary flow of the patient improved to TIMI grade 3 (Fig. 4A, B). The period from his arrival to the stent implantation was 35 minutes. The patient was directly transferred to the operating room for surgical repair of the aortic dissection without computed tomography. Aortotomy revealed the entry of the dissection existed in the ascending aorta, extendeding to the sinus of Valsalva. The implanted stent sealed the dissecting hematoma tightly, extending to the orifice of the LMT (Fig. 5). Thereafter, replacement of the ascending aorta was performed. Because the patient’s hemodynamics were particularly unstable, he left the operation room under PCPS, with intra-aortic balloon pumping in place. He could not be weaned from cardiopulmonary support afterwards, and died on his seventh day of hospitalization.

## Discussion

Acute myocardial infarction (AMI) combined with aortic dissection occasionally occured. In Japanese population, the frequency of this condition among dissection patients has been reported to be 6.1-7.7% [1, 2]. It may arise from the obstruction of the ostium of the coronary artery by the intimal flap, or from the extension of the dissection into the coronary artery itself, resulting in the associated luminal compression by dissecting hematoma [3]. Aortic dissection involving the left coronary artery occurs less frequently but is more lethal than that involving the right coronary artery. It is difficult to make a differential diagnosis between a simple AMI and the infarction resulting from the ascending aortic dissection. Even when the electrocardiographic evidence indicates the presence of a myocardial ischemia, the possibility of an aortic dissection should not be ignored. The diagnosis of a simple AMI may lead to the inappropriate administration of thrombolytic or anticoagulant treatment, resulting in catastrophic consequences. Although surgical outcomes of aortic dissections have improved, the mortality rate of aortic dissection complicated with AMI remains high. Therefore, it is essential to obtain a prompt and correct diagnosis and to proceed with an appropriate treatment.

Contrast-enhanced computed tomography (CT) scan is one of the most useful imaging methods to establish the diagnosis of aortic dissection. However, in the case of a patient with aortic dissection combined with AMI, it may be impossible to perform the CT scan because the patient is often under unstable hemodynamic conditions. Although TTE could be performed expeditiously at bedside, TTE sensitivity in detecting aortic dissection is lower than that when a CT scan is used [4]. In our case, it was in fact difficult to make an accurate diagnosis of aortic dissection before the CAG. We could not perform the TTE so carefully as to idenify the dissection flap, because we needed to move to catheralization room as soon as possible for the patient’s unstable hemodynamics. The IVUS imaging revealed a intimal flap was fluctuating and a large intramural hematoma compressed the ostium of the left main trunk. This distinctive image brought the correct diagnosis of ascending aortic dissection involving the coronary artery.

To save patients when such life-threatening conditions are involved, aggressive coronary revascularization to prevent extensive myocardial damage is essential. Although angioplasty for unprotected LMT has been controversial, it would be important to restore the coronary circulation as quickly as possible in case of acute obstruction of LMT. Several cases have been reported in which percutaneous coronary angioplasty was performed preoperatively. Some authors reported the placement of a perfusion catheter into the dissected coronary artery [5, 6], while others reported treating the dissected coronary artery with stent implantation [7-9]. Both procedures could improve patient hemodynamic status by maintaining an adequate coronary perfusion. These techniques as a bridge to surgery could gain valuable time for the critically unstable patients before definitive surgery takes place.

In our case, unfortunately, the infarcted myocardium could not recover in spite of the relatively short period of time from the arrival on our hopital to the angioplasty. Aortic dissection involving the left main trunk of the coronary artery rarely occurs, but results in extremely serious conditions. Therefore, a timely and correct diagnosis is very important. When electrocardiogram reveales ST-segment change, the patient may be transferred directly to catheterization laboratory for coronary angiography. Furthermore it is possible that the presence of an aortic dissection is overlooked even after percutaneous coronary intervention. In the present case, we also could not obtain an accurate diagnosis before the angiography, but we finally succeeded in diagnosing the condition by use of intravascular ultrasound.
